# Frequency of Post-Treatment Recurrence After Short-Course Regimens for Drug-Resistant Tuberculosis: A Systematic Review and Meta-Analysis

**DOI:** 10.3390/jcm15218273

**Published:** 2026-10-23

**Authors:** Roman S. Ladyu, Anastasia I. Gaida, Viktor A. Mudrov, Platon I. Eliseev, Ruslan O. Khachetlov, Ksenia V. Rudakova, Valentina V. Tinkova, Anastasia G. Samoilova, Irina A. Vasilyeva

**Affiliations:** 1 Federal State Budgetary Institution “National Medical Research Center for Phthisiopulmonology and Infectious Diseases” of the Ministry of Health of the Russian Federation, 4 Dostoevskogo St., Bldg. 2, Moscow 127473, Russia; nsovca@yandex.ru (A.I.G.); eliseevpi@nmrc.ru (P.I.E.); ballayana1@mail.ru (R.O.K.); rudakova_kv@nmrc.ru (K.V.R.); tinkovavv@nmrc.ru (V.V.T.); a.samoilova.nmrc@mail.ru (A.G.S.); nmrc@nmrc.ru (I.A.V.); 2 Chita State Medical Academy, Ministry of Health of the Russian Federation, 39A Gorkogo St., Chita 672000, Russia; mudrov_viktor@mail.ru

**Keywords:** drug-resistant tuberculosis, multidrug-resistant tuberculosis, short-course treatment, relapse, recurrence, systematic review, meta-analysis, person-years

## Abstract

**Background/Objectives**: Short-course regimens (SCRs) are increasingly used to treat drug-resistant tuberculosis (DR-TB), but data on post-treatment recurrence remain limited. We aimed to estimate the frequency of post-treatment recurrence after SCRs and compare recurrence between SCRs and comparator regimens. **Methods**: We conducted a systematic review and meta-analysis of clinical studies reporting treatment duration, post-treatment follow-up, and recurrent tuberculosis episodes after SCRs for DR-TB. The primary single-arm analysis used a binomial-normal random-effects generalized linear mixed model (GLMM) based on r/A, retaining zero-event cohorts without a continuity correction. Comparative analyses used pooled risk ratios (RRs) and person-time-adjusted incidence rate ratios (IRRs). **Results**: Twenty-five studies contributed 26 SCR cohorts. In the primary analysis, 98 recurrent tuberculosis episodes occurred among 6954 patients; the pooled proportion was 1.24% (95% confidence interval [CI], 0.79–1.93%; 95% prediction interval, 0.24–6.21%). The pooled proportion was 1.20% (95% CI, 0.70–2.03%) for fully oral SCRs, 1.57% (0.85–2.85%) for pretomanid-containing SCRs, 1.55% (0.93–2.56%) for SCRs lasting ≤ 39 weeks, and 1.95% (1.20–3.15%) for SCRs lasting ≤ 36 weeks. In the overall comparative analysis, no statistically significant difference in post-treatment recurrence was observed between SCRs and comparator regimens: the pooled RR was 1.35 (95% CI, 0.34–5.40), and the person-time-adjusted IRR was 1.25 (95% CI, 0.50–3.15). In the randomized controlled trial (RCT) subset, the pooled RR indicated a higher recurrence risk with SCRs (RR, 2.72; 95% CI, 1.25–5.89), whereas the corresponding person-time-adjusted estimate did not show a statistically significant difference (IRR, 1.66; 95% CI, 0.81–3.43). This divergence indicates that comparative estimates are sensitive to how post-treatment follow-up duration is accounted for. Meta-regression identified no statistically significant associations between recurrence frequency and the examined cohort-level clinical characteristics. The certainty of evidence, assessed using the Grading of Recommendations Assessment, Development and Evaluation (GRADE) framework, was low for the estimated recurrence frequency after fully oral SCRs, very low for the subgroup estimates, and low for the comparative estimates. **Conclusions**: These findings indicate a low frequency of post-treatment tuberculosis recurrence after SCRs and support the durability of treatment outcomes achieved with these regimens. However, substantial between-study heterogeneity and differences in post-treatment follow-up limit the interpretation of the pooled estimate as a uniform long-term recurrence risk. Further studies with standardized follow-up beginning at treatment completion are needed to provide more precise estimates of long-term outcomes after SCRs.

## 1. Introduction

Treatment strategies for drug-resistant tuberculosis (DR-TB) aim to improve treatment efficacy, safety, and adherence. The introduction of new anti-tuberculosis drugs has enabled the development of shorter, fully oral treatment regimens [1,2]. In 2022, the World Health Organization (WHO) updated its recommendations for patients with rifampicin-resistant tuberculosis (RR-TB), including multidrug-resistant tuberculosis (MDR-TB), recommending new shorter treatment regimens [1]. As treatment duration is shortened, evaluation should extend beyond treatment completion to determine whether treatment success is sustained and to quantify tuberculosis recurrence during post-treatment follow-up [1,2,3,4].

Evidence on post-treatment recurrence after short-course regimens has expanded substantially in recent years. A previously published meta-analysis of shorter, fully oral regimens included studies available through November 2023 [5]. Since then, additional randomized trials, cohort studies, and extended follow-up data have become available across a broad range of short-course regimens [6,7,8,9,10,11,12,13,14,15,16,17,18,19,20,21,22,23,24,25,26,27,28,29,30]. These studies provide additional information on the durability of treatment outcomes after completion of therapy. Baseline disease extent, drug penetration and regimen activity, treatment adherence, and other clinical factors may contribute to the risk of post-treatment recurrence [31,32,33]. An updated synthesis is therefore needed to incorporate these data and evaluate recurrence across a broader range of short-course regimens.

### 1.1. Objective

To conduct a systematic review and meta-analysis of clinical study data on the frequency of post-treatment tuberculosis recurrence following short-course regimens in patients with RR-TB, MDR-TB, pre-extensively drug-resistant tuberculosis (pre-XDR-TB), and extensively drug-resistant tuberculosis (XDR-TB).

### 1.2. Study Objectives

To systematically identify and select published clinical studies reporting recurrent tuberculosis episodes after short-course treatment regimens for DR-TB.

To extract data on treatment regimens, the presence of a comparison group, the drug-resistance profile of M. tuberculosis, the number of patients whose post-treatment outcomes were assessed, the duration of follow-up, and the number of recorded recurrent tuberculosis episodes.

To assess the frequency of post-treatment tuberculosis recurrence after short-course regimens (SCRs) for DR-TB.

To compare post-treatment tuberculosis recurrence after SCRs and comparator regimens (CRs) for DR-TB.

## 2. Materials and Methods

The review was registered in Open Science Framework (OSF) Registries [34], digital object identifier (DOI): 10.17605/OSF.IO/4NUK5, and was reported in accordance with the Preferred Reporting Items for Systematic Reviews and Meta-Analyses Supplementary Material (PRISMA) 2020 Checklist [35]. A systematic search was conducted in PubMed and Europe PMC. ClinicalTrials.gov, Chinese Clinical Trial Registry (ChiCTR), and Russian-language sources were also searched. Embase, Web of Science, Scopus, and CENTRAL were not searched. The systematic search covered publications from 1 January 2018 through 1 May 2026. Before finalization of the revised manuscript, a targeted update was performed to identify final publications and extended follow-up reports from studies already identified through the systematic search, trial registries, or related reports. The key search concept blocks are presented in Table 1.

### 2.1. Study Selection Process

Search queries were adapted to the syntax of each database. 

The search considered publication type (clinical studies, randomized controlled trials, and cohort studies) and language (English, Russian, and Chinese). Reference lists of included publications and previously published reviews of short-course treatment regimens for DR-TB were also screened.

Search results, including the publication title, abstract, keywords, authors, publication year, journal, and DOI, were exported into a standardized table.

A Python script (Python 3.13) was used to remove duplicates and flag potentially irrelevant records for preliminary review. The term that triggered each flag was retained to allow verification of the filtering process. The final decision to exclude a publication was made after assessing it against the eligibility criteria.

### 2.2. Inclusion Criteria

Studies of short-course treatment regimens for DR-TB in patients of any age.

Clinical studies of short-course treatment regimens for DR-TB:

- Randomized comparative and single-arm studies;
- Non-randomized comparative studies;
- Prospective and retrospective cohort studies.

Full-text journal articles or full-text preprints of completed studies reporting data on treatment duration, post-treatment follow-up, and recurrent tuberculosis episodes.

### 2.3. Exclusion Criteria

Studies with an analytical cohort of fewer than 20 patients in whom recurrent tuberculosis episodes were assessed after treatment completion.

Insufficient information to establish that recurrent tuberculosis was assessed after treatment completion and/or to determine the size of the population in which this outcome was assessed.

**Figure 1:**
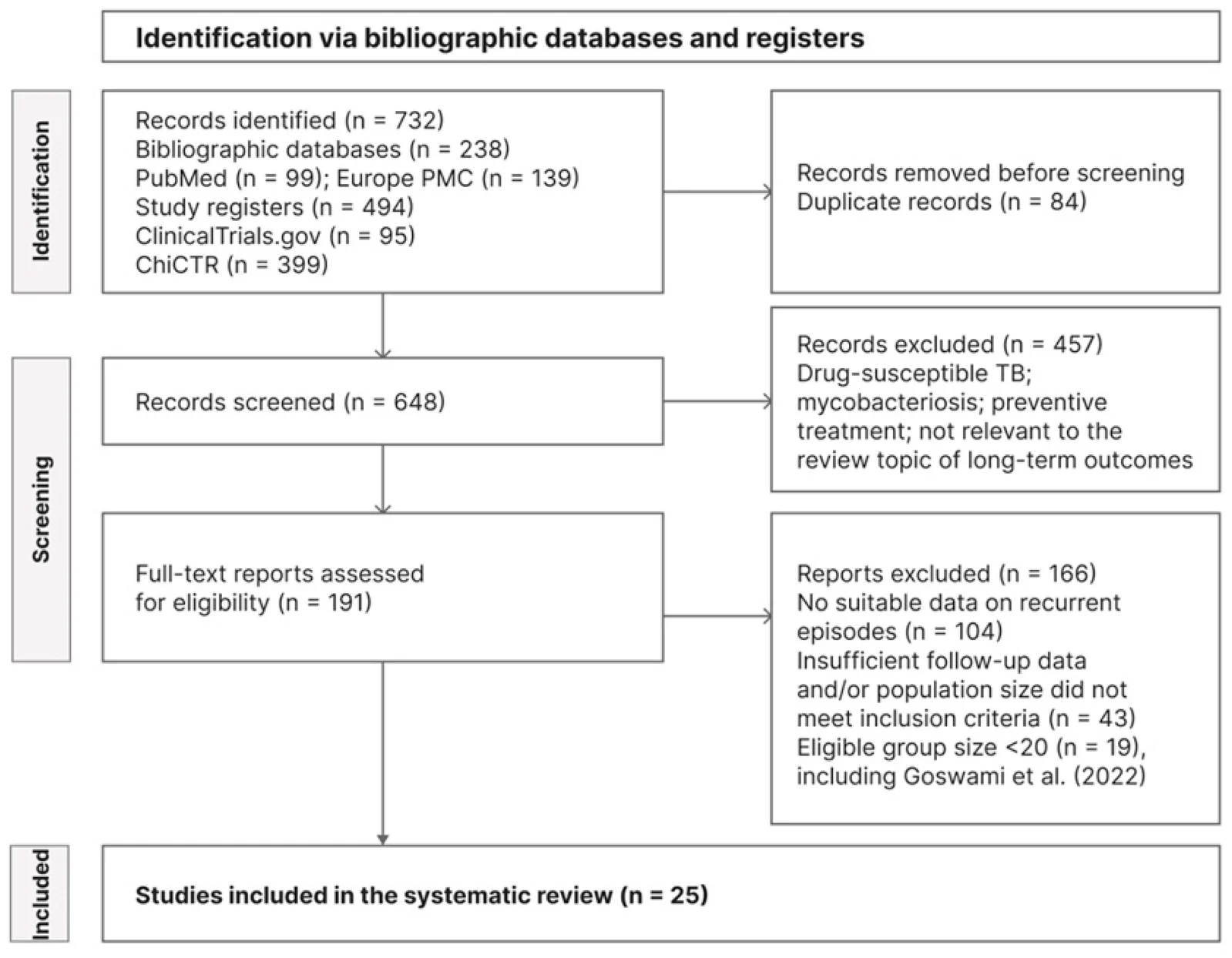
Study identification, screening, and inclusion in the systematic review [35,36].

After exclusion of publications that did not meet the eligibility criteria, 25 studies were included in the systematic review (Figure 1).

### 2.4. Data Extraction

For each study, the following data were extracted from the primary publication and related materials:

- Authors, year of publication, and country or countries in which the study was conducted;
- Study design, number of analyzed groups, and presence of a comparison group;
- The drug-resistance profile of M. tuberculosis, composition of the anti-tuberculosis regimen, planned treatment options, and treatment modifications. In comparative studies and studies with multiple groups, data for each included regimen and comparison group were extracted separately;
- The number of patients enrolled in the study and in each analyzed group before exclusions from the outcome analysis. The overall study population and the initial size of each individual group were recorded separately;
- The number of recurrent tuberculosis episodes recorded after successful treatment completion, denoted as r;
- N, representing the source-reported size of the corresponding treatment group or analysis population to which the extracted number of recurrent tuberculosis episodes (r) referred, before restriction to the post-treatment analytical denominator A. When several source populations were reported, the population corresponding to the selected treatment arm and extracted event count was used;
- Treatment duration for each analyzed group, including the planned duration, median, mean, or range of actual treatment durations;
- Duration of post-treatment follow-up;
- Clinical and microbiological characteristics, including baseline sputum smear positivity, cavitary or destructive lung changes, human immunodeficiency virus (HIV) infection, and diabetes mellitus. For each characteristic, the number of patients with that characteristic and the corresponding number with available data were extracted.

To standardize the published data, additional calculations were performed for studies in which the number of recurrent tuberculosis episodes among patients with a favorable treatment outcome was not reported. For the r/A calculation, the value of A corresponded to the number of patients with a successful treatment outcome included in the post-treatment recurrence analysis according to the original study. Patients were included in this population according to the criteria used by the study authors. Successful treatment included the categories “cured,” “treatment completed,” and their author-defined equivalents, including “effective course of chemotherapy.” If the number of patients who successfully completed the full treatment course could not be determined unambiguously from the published data, A was not reconstructed.

Study selection was performed by two authors. Automated filtering was used for the preliminary identification of potentially irrelevant records and did not constitute an independent basis for the final exclusion of publications. Data extraction was performed by one author, and the extracted data were subsequently independently verified by other authors.

In the included studies, recurrent tuberculosis was reported using the terms relapse, recurrence, disease recurrence, recurrent TB, recurrence after success, bacteriologic relapse, and relapse/reinfection. Recurrent tuberculosis episodes were extracted according to the definitions and classifications provided in the source publications. When additional clinical, microbiological, or molecular information was available, it was used to classify the event. Episodes identified as reinfection with a different M. tuberculosis strain or as events not confirmed as recurrent tuberculosis, were excluded from the primary event count. When relapse and reinfection could not be distinguished, the episode was retained as recurrent tuberculosis of undetermined origin. A sensitivity analysis included all post-treatment recurrence events reported by the original study authors [10,18,21,27,28].

### 2.5. Risk of Bias Assessment of the Included Studies

Risk-of-bias assessments were performed by one reviewer and independently verified by a second reviewer; disagreements were resolved through discussion and consensus. Risk of bias was assessed according to study design. Risk of Bias 2 (RoB 2) [37] was used for randomized controlled trials (Table 2), Risk Of Bias In Non-randomized Studies of Interventions (ROBINS-I) [38] for non-randomized comparative studies (Table 3), and an adapted Newcastle–Ottawa Scale [39] for single-arm cohort studies (Table 4). For single-arm cohorts, the adapted Newcastle–Ottawa assessment comprised six prespecified domains: cohort definition, regimen data, post-treatment recurrence, outcome assessment, follow-up period, and follow-up completeness; one point was assigned for each criterion met.

The certainty of evidence for the main findings was assessed using the Grading of Recommendations Assessment, Development and Evaluation (GRADE) framework [40], taking into account risk of bias, inconsistency, indirectness, imprecision, and potential publication bias.

### 2.6. Selection of Groups for Meta-Analysis

For the endTB [8] and TB-PRACTECAL [10] randomized controlled trials, the groups for quantitative synthesis were selected separately for each trial. In endTB [8], the three short-course regimens recommended by WHO were included. In TB-PRACTECAL, the BPaLM (bedaquiline, pretomanid, linezolid, and moxifloxacin) intervention group and the corresponding control group were used as the primary comparison for the regimen selected for the second stage of the trial and reported in the TB-PRACTECAL publication [10]. The BPaLC (bedaquiline, pretomanid, linezolid, and clofazimine) and BPaL (bedaquiline, pretomanid, and linezolid) groups, whose results were presented in the selected publication as additional post hoc analyses, were not included.

For Ndjeka et al. [15], the fully oral short-course regimen group was included in the single-arm analysis. In STREAM Stage 2 [13], the 6-month and 9-month experimental groups were considered separately in the single-arm meta-analysis. STREAM Stage 2 [13] was not included in the comparative meta-analysis because it would contribute more than one comparison from the same multi-arm trial, creating within-study dependence; no single experimental comparison was selected arbitrarily. For Le T.N. Anh et al. [29], the 2016 subgroup was used, comprising 99 patients who initiated treatment and 79 who successfully completed treatment and were assessed after treatment completion.

### 2.7. Statistical Analysis

1. Single-arm meta-analysis (Table 5);
2. Comparative meta-analysis of SCRs and comparator regimens (Table 6);
3. Exploratory meta-regression of cohort-level clinical characteristics.

Two random-effects approaches were used to estimate the proportion of recurrent tuberculosis episodes. The primary pooled estimate was obtained using a binomial-normal generalized linear mixed model (GLMM) with a logit link and maximum-likelihood estimation using adaptive Gauss–Hermite quadrature with 7 quadrature points (nAGQ = 7) under a random-effects model [41]. Study-specific 95% confidence intervals were calculated using the exact Clopper–Pearson method. This model allowed zero-event cohorts to be included without applying a continuity correction. In the inverse-variance logit meta-analysis, only cohorts with at least one recurrent tuberculosis episode were included, and between-study variance (τ^2^) was estimated using restricted maximum likelihood (REML). The primary single-arm analyses were based on r/A. As a denominator sensitivity analysis, the same models were repeated using r/N [42].

An additional outcome-definition sensitivity analysis repeated the primary single-arm GLMM using the number of post-treatment recurrence events reported by the original study authors instead of the event count used in the primary analysis, in which confirmed reinfections were excluded when molecular typing allowed this distinction. For the GLMM analyses, between-study heterogeneity was summarized using τ^2^ and I^2^ and assessed using a likelihood-ratio test. A 95% prediction interval was calculated on the logit scale and back-transformed to the proportion scale.

For eligibility and subgroup assignment, regimen duration was based on the nominal planned duration reported by the source. A short-course regimen was eligible if its nominal planned duration was no more than 12 months. Thresholds of ≤39 and ≤36 weeks were used for additional analyses of shorter regimens; ≤39 weeks approximately corresponds to a 9-month course, whereas ≤36 weeks defines a narrower group within this category. When duration was reported in months, conversion to weeks in Table 7 was descriptive only and did not alter the eligibility classification.

A regimen was considered fully oral if it did not include injectable anti-tuberculosis drugs at any point during the analyzed treatment course.

The incidence rate of recurrent tuberculosis episodes was expressed per 100 person-years of follow-up: IR = r/PY × 100. When individual or aggregate person-time at risk was not reported, person-years were approximated as PY = A × T/52, where T denotes the duration of post-treatment follow-up in weeks and a year was assumed to contain 52 weeks. These estimates were interpreted as approximate incidence rates rather than individual time-to-recurrence estimates.

#### Comparative Meta-Analysis

Comparative analyses used two complementary effect measures. The primary comparative analysis used the risk ratio (RR) based on r/A in each treatment arm. A person-time-adjusted incidence rate ratio (IRR) was used as an additional comparative analysis to account for differences in the duration of post-treatment follow-up between groups. The primary comparative set included all eligible comparative studies. The RR and person-time-adjusted IRR analyses were additionally repeated in the randomized controlled trial (RCT) subset.

In the primary comparative meta-analysis, risk ratios (RRs) were calculated using r/A. As a denominator sensitivity analysis, the calculation was repeated using r/N. Pooled RRs were estimated using a random-effects model with between-study variance estimated by restricted maximum likelihood (REML) and the Hartung–Knapp adjustment. When events were absent in only one of the compared arms, a continuity correction of 0.5 was applied to both arms. Studies with no events in either arm were retained in the source dataset but did not contribute to the pooled RR estimate.

For each treatment group, the incidence rate of recurrent tuberculosis episodes after treatment completion was first calculated as the number of recorded episodes (r) divided by the total post-treatment person-time in person-years (PY). The incidence rate in the short-course regimen group was then divided by the corresponding incidence rate in the comparator group:

IRR = (rSCR/PYSCR)/(rCR/PYCR).

Therefore, IRR < 1 indicates a lower incidence rate of recurrent tuberculosis episodes with the short-course regimen, whereas IRR > 1 indicates a higher incidence rate compared with the comparator group.

The person-time-adjusted IRR analysis included comparative studies for which post-treatment follow-up duration (T) could be established for both treatment arms. Study-specific IRRs were pooled using a random-effects model with between-study variance estimated by restricted maximum likelihood (REML) and the Hartung–Knapp adjustment. When events were absent in only one of the compared arms, a continuity correction of 0.5 was applied to both arms. Comparisons with no events in either arm did not contribute to the pooled IRR estimate. The study by Maryandyshev et al. [14] was not included in the IRR analysis because a single post-treatment follow-up duration could not be established from the published data. The endTB trial [8] was retained in the source dataset but did not contribute an estimable IRR because no events occurred in either arm.

To assess possible sources of between-study heterogeneity, separate univariable binomial-normal GLMM meta-regression analyses were performed using aggregate cohort-level data. The proportions of patients with cavitary or destructive lung changes, baseline sputum smear positivity, HIV infection, and diabetes mellitus were used as covariates. Effects were expressed as odds ratios (ORs) per 10-percentage-point increase in the corresponding covariate. Meta-regression was considered exploratory. The absence of a statistically significant association was not interpreted as evidence of the absence of a clinically meaningful effect at the individual level.

Small-study effects were assessed in the primary single-arm dataset using a binomial-normal GLMM meta-regression with 1/√A as the predictor. Additional exploratory assessments among the 20 cohorts with at least one event under the primary outcome definition included a random-effects regression test using 1/√A, a standard error (SE)-based Egger-type regression test, Kendall’s rank-correlation test, and visual inspection of a funnel plot. These analyses were interpreted as assessments of small-study effects or funnel-plot asymmetry and not as proof of publication bias.

Sensitivity analyses were performed to assess the robustness of the comparative estimates. Leave-one-out analyses were performed separately for the primary RR analysis based on r/A, the sensitivity RR analysis based on r/N, and the person-time-adjusted IRR analysis by sequentially excluding each study from the corresponding analysis set and refitting the same random-effects model. For the IRR analysis, sensitivity to assumptions used to reconstruct post-treatment follow-up time (T) was additionally evaluated using alternative T values for MDR-END [7], TB-PRACTECAL [10], and Ndjeka et al. [15], as well as by excluding BEAT TB [9]. Scenarios yielding zero post-treatment follow-up time were not used to estimate IRR.

Statistical analyses were performed in R version 4.6.0 using the metafor package version 5.0-1 [44].

As an external benchmark, we used the previously published meta-analysis by Yosofi et al. [5], in which the pooled relapse proportion among 1792 patients receiving fully oral short-course regimens for DR-TB was 2.0% (95% confidence interval (CI), 1.0–3.0%).

## 3. Results

### 3.1. General Characteristics of the Included Studies

Twenty-five studies contributing 26 analytical short-course regimen cohorts were included in the systematic review (Table 7). The comparative analytical set included nine studies [6,7,8,9,10,11,12,14,15], represented by nine pairs of treatment groups, including seven RCTs [6,7,8,9,10,11,12] and two non-randomized comparative studies [14,15]. The primary single-arm analysis included 26 cohorts from 25 studies. 

The clinical characteristics of the included cohorts are presented in Table 8.

#### 3.1.1. Baseline Sputum Smear Positivity

The proportion of patients with baseline sputum smear positivity varied substantially across studies, ranging from 30.4% in MDR-END [7] to 99.0% in STREAM Stage 2 (9 months) [13]. High proportions were also reported in STREAM Stage 2 (6 months) [13], 97.8%; TRUST-plus [26], 97.5%; STREAM 1 [6], 96.8%; BEAT-India [23], 95.2%; and Daniel B.D. [27], 92.3%.

#### 3.1.2. Cavitary or Destructive Lung Changes

The proportion of patients with cavitary or destructive lung changes ranged from 18.2% in BEAT TB [9] to 84.4% in Nix-TB [16]. The highest proportions were reported in Nix-TB [16], 84.4%; TRUST-plus [26], 82.5%; STREAM 1 [6], 77.0%; STREAM Stage 2 (9 months) [13], 75.5%; STREAM Stage 2 (6 months) [13], 75.0%; Golubchikov P.N. [30], 69.0%; Khimova E.S. [20], 68.3%; endTB-Q [11], 66.3%; ZeNix [19], 61.9%; endTB trial [8], 59.2%; Korotych [17], 57.7%; TB-PRACTECAL [10], 55.1%; NExT trial [12], 53.1%; and BEAT-India [23], 50.9%.

#### 3.1.3. HIV Infection

Among studies with available data, the highest proportion of patients with HIV infection was reported in Ndjeka [15], 71.7%; followed by NExT trial [12], 55.1%; BEAT TB [9], 51.7%; Nix-TB [16], 51.4%; STREAM 1 [6], 33.6%; Golubchikov P.N. [30], 26.2%; TB-PRACTECAL [10], 24.6%; Fardhdiani [21], 23.0%; ZeNix [19], 19.9%; and Schwœbel [18], 19.9%. No HIV infection was reported in MDR-END [7] or TRUST-plus [26] (0.0%). Among studies with non-zero values, the lowest proportions were reported by Daniel B.D. [27], 0.5%, and in endTB-Q [11], 0.6%. HIV data were not reported in BEAT-India [23].

#### 3.1.4. Diabetes Mellitus

The highest proportion of patients with diabetes mellitus was reported in Burhan E. [24], 36.9%; followed by endTB-Q [11], 22.7%; MDR-END [7], 20.3%; BIG cohort [25], 20.0%; and endTB trial [8], 16.3%. Lower proportions were reported by Daniel B.D. [27], 12.2%; BEAT-India [23], 11.5%; STREAM Stage 2 (6 months) [13], 9.8%; Sinha [22], 8.9%; TRUST-plus [26], 8.8%; Khimova E.S. [20], 8.4%; STREAM 1 [6], 8.3%; Korotych [17], 8.3%; STREAM Stage 2 (9 months) [13], 8.2%; ZeNix [19], 5.0%; and Fardhdiani [21], 4.3%.

### 3.2. Results of the Single-Arm Meta-Analysis

The primary single-arm analysis included 26 cohorts from 25 studies, with 98 recurrent tuberculosis episodes recorded among 6954 patients in the post-treatment analytical population. STREAM Stage 2 [13] contributed two separate short-course regimen cohorts; the publication was counted once when determining the number of studies. The analysis of fully oral SCRs included 20 cohorts, with 74 recurrent tuberculosis episodes among 5532 patients. In the subgroup of pretomanid-containing SCRs, 21 recurrent tuberculosis episodes were recorded among 1260 patients in 7 cohorts. The analysis of SCRs with a nominal duration of ≤39 weeks included 16 cohorts, with 70 recurrent tuberculosis episodes among 4594 patients; the analysis of SCRs with a nominal duration of ≤36 weeks included 11 cohorts, with 37 recurrent tuberculosis episodes among 1747 patients. Summary descriptive data are presented in Table 9.

**Figure 2:**
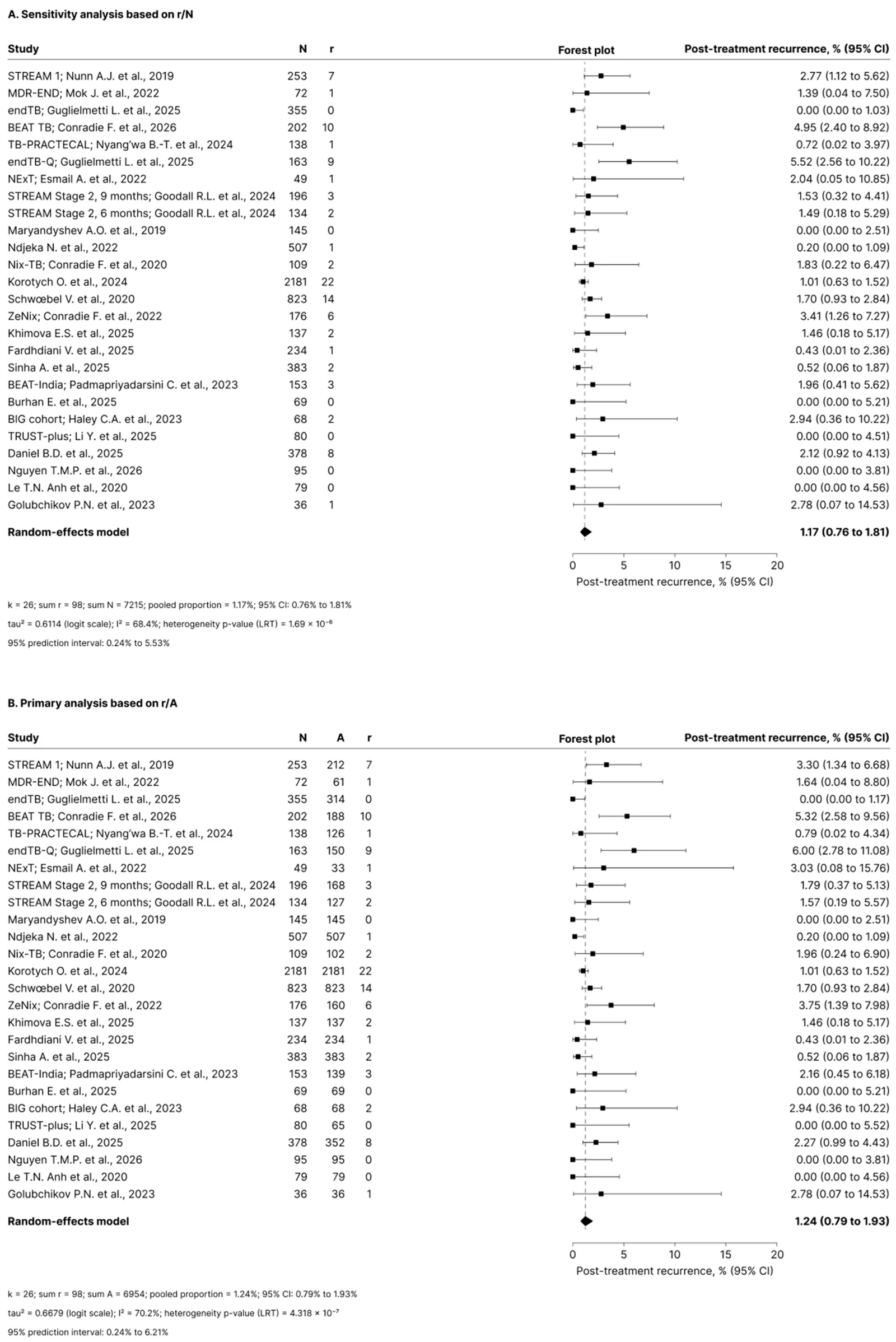
Forest plots of post-treatment tuberculosis recurrence after short-course regimens. (**A**) Denominator sensitivity analysis based on r/N; (**B**) Primary analysis based on r/A [6,7,8,9,10,11,12,13,14,15,16,17,18,19,20,21,22,23,24,25,26,27,28,29,30].

In the GLMM analysis of all SCRs lasting ≤ 12 months, the pooled proportion of recurrent tuberculosis episodes was 1.24% (95% CI, 0.79–1.93%). Between-study heterogeneity was substantial (I^2^ = 70.2%; *p* < 0.001), and the 95% prediction interval was 0.24–6.21%. The 95% CI reflects the uncertainty of the pooled estimate, whereas the prediction interval represents the expected range of the true proportion of recurrent tuberculosis episodes in a new comparable cohort after accounting for between-study variability. Because the duration of post-treatment follow-up differed across studies, the pooled proportion should not be interpreted as the risk of a recurrent tuberculosis episode over a single fixed time period. In the denominator sensitivity analysis using N, the pooled proportion was 1.17% (95% CI, 0.76–1.81%), which did not materially change the primary estimate (Figure 2).

In the analysis of fully oral SCRs, the pooled proportion of recurrent tuberculosis episodes was 1.20% (95% CI, 0.70–2.03%). Between-study heterogeneity was substantial (I^2^ = 72.5%; *p* < 0.001), and the 95% prediction interval was 0.20–6.95%. Despite the pooled estimate of 1.20%, the wide 95% prediction interval indicates substantial variability in the expected proportion of recurrent tuberculosis episodes across comparable cohorts. When N was used as the denominator, the pooled proportion was 1.13% (95% CI, 0.67–1.89%).

In the subgroup of pretomanid-containing SCRs, the pooled proportion of recurrent tuberculosis episodes was 1.57% (95% CI, 0.85–2.85%). Between-study heterogeneity was less pronounced (I^2^ = 36.0%; *p* = 0.070), and the 95% prediction interval was 0.53–4.53%. Interpretation should take into account the limited number of cohorts and recorded events: the estimate was based on 7 cohorts and 21 recurrent tuberculosis episodes. This estimate applies to pretomanid-containing regimens as a group and does not allow the independent effect of pretomanid to be assessed. When N was used as the denominator, the pooled proportion was 1.50% (95% CI, 0.84–2.68%).

For SCRs with a nominal duration of ≤39 weeks, the pooled proportion of recurrent tuberculosis episodes was 1.55% (95% CI, 0.93–2.56%). Between-study heterogeneity was substantial (I^2^ = 69.2%; *p* < 0.001), and the 95% prediction interval was 0.33–7.04%. The wide prediction interval indicates that the proportion of recurrent tuberculosis episodes in individual comparable cohorts may differ substantially from the pooled estimate. In the denominator sensitivity analysis using N, the pooled proportion was 1.45% (95% CI, 0.88–2.38%).

For SCRs with a nominal duration of ≤36 weeks, the pooled proportion of recurrent tuberculosis episodes was 1.95% (95% CI, 1.20–3.15%). Between-study heterogeneity was moderate (I^2^ = 43.6%; *p* = 0.030), and the 95% prediction interval was 0.66–5.64%. In the denominator sensitivity analysis using N, the pooled proportion was 1.83% (95% CI, 1.14–2.93%). The point estimate was numerically higher than that for SCRs lasting ≤ 39 weeks; however, the ≤36-week subgroup is nested within the broader ≤39-week subgroup. Therefore, the difference between these estimates does not constitute an independent comparison and does not establish an association between shorter treatment duration and an increased proportion of recurrent tuberculosis episodes. The corresponding pooled GLMM results are summarized in Table 10.

In the additional two-stage logit meta-analysis, zero-event cohorts were excluded without applying a continuity correction. For all SCRs, the pooled proportion of recurrent tuberculosis episodes based on A was 2.02% (95% CI, 1.42–2.85%). In the fully oral and pretomanid-containing SCR subgroups, the corresponding estimates were 1.91% (95% CI, 1.24–2.96%) and 2.03% (95% CI, 1.18–3.49%), respectively; for SCRs lasting ≤39 and ≤36 weeks, the estimates were 2.25% (95% CI, 1.45–3.46%) and 2.42% (95% CI, 1.56–3.75%), respectively (Table 11). These estimates were higher than the corresponding GLMM estimates; the difference may reflect both the statistical method and the exclusion of cohorts with no recorded events.

In the outcome-definition sensitivity analysis, use of all post-treatment recurrence events reported by the original study authors increased the number of events from 98 to 109 while the analytical denominator remained unchanged at A = 6954. The pooled proportion was 1.43% (95% CI, 0.95–2.16%; I^2^ = 69.4%), compared with 1.24% (95% CI, 0.79–1.93%; I^2^ = 70.2%) in the primary analysis. Differences in event counts occurred in five cohorts [10,18,21,27,28]. Broadening the outcome definition resulted in a modest increase in the pooled point estimate.

### 3.3. Results of the Comparative Meta-Analysis

**Figure 3:**
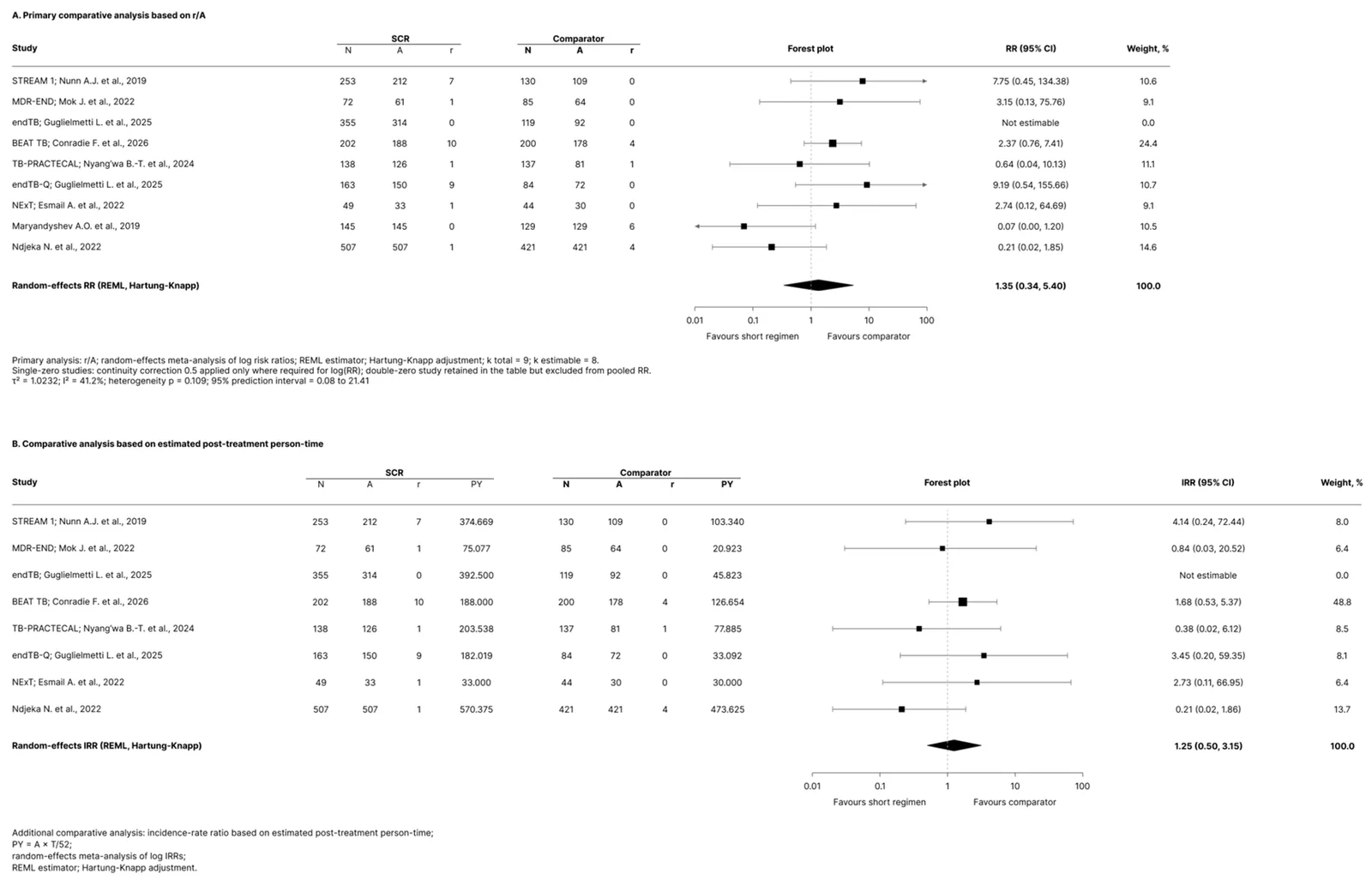
Comparative meta-analysis of post-treatment tuberculosis recurrence after short-course versus comparator regimens. (**A**) Risk ratios based on the post-treatment analytical denominator A; (**B**) Incidence rate ratios based on estimated post-treatment person-time [6,7,8,9,10,11,12,14,15,43].

Given the low pooled recurrence frequency observed in the single-arm analysis, we next examined whether post-treatment recurrence differed between SCRs and the corresponding comparator regimens. The primary comparative analysis included nine studies. Across these studies, 30 recurrent tuberculosis episodes were recorded among 1736 patients in the post-treatment analytical population of the SCR groups, compared with 15 episodes among 1176 patients in the comparator groups. In the full comparative set, the pooled RR based on r/A was 1.35 (95% CI, 0.34–5.40; I^2^ = 41.2%), showing no statistically significant difference in recurrence risk between SCRs and comparator regimens (Figure 3A). Repeating the analysis with the original group population N as the denominator produced a similar estimate (RR, 1.45; 95% CI, 0.36–5.83; I^2^ = 41.4%), indicating that the overall conclusion was not sensitive to the choice of denominator.

Accounting for the duration of post-treatment follow-up yielded a consistent overall finding. Among the eight studies with available person-time, seven contributed an estimable IRR; 30 recurrent episodes occurred over an estimated 2019.18 person-years in the SCR groups and 9 over 911.34 person-years in the comparator groups. The pooled IRR was 1.25 (95% CI, 0.50–3.15; I^2^ = 0%), again showing no statistically significant difference between groups (Figure 3B).

In the RCT subset, however, the pooled RR based on r/A was higher at 2.72 (95% CI, 1.25–5.89; I^2^ = 0%), with a similar result when N was used as the denominator (RR, 2.95; 95% CI, 1.49–5.86; I^2^ = 0%). When post-treatment person-time was taken into account in the same RCT subset, the pooled IRR was 1.66 (95% CI, 0.81–3.43; I^2^ = 0%) and its confidence interval included 1. Thus, the comparative result differed according to whether recurrence was analyzed as a cumulative risk or as an incidence rate accounting for post-treatment follow-up time. Detailed comparative estimates are presented in Table 12.

#### Leave-One-out Analyses and Sensitivity to Follow-Up Duration

Sequential leave-one-out analyses showed that the pooled RR based on r/A ranged from 1.07 after exclusion of endTB-Q to 1.94 after exclusion of Ndjeka, compared with 1.35 (95% CI, 0.34–5.40) in the full analysis. For r/N, the corresponding range was 1.16–2.16, compared with the full-model estimate of 1.45 (95% CI, 0.36–5.83). The pooled IRR ranged from 0.96 after exclusion of BEAT TB to 1.66 after exclusion of Ndjeka, compared with 1.25 (95% CI, 0.50–3.15) in the full analysis; the 95% CI included 1 in all leave-one-out analyses. When alternative values for the duration of post-treatment follow-up were used for MDR-END, TB-PRACTECAL, and Ndjeka, the pooled IRR ranged from 1.18 to 1.29. After exclusion of BEAT TB, the IRR was 0.96 (95% CI, 0.24–3.91). In all examined scenarios, the 95% CI for the pooled IRR included 1.

### 3.4. Comparison with Previously Published Data on Short-Course Regimens

The previously published meta-analysis by Yosofi et al. [5] included 10 studies published through November 2023 and focused on relapse after fully oral short-course treatment regimens for DR-TB among patients with favorable treatment outcomes. In that study, the pooled relapse proportion was 2.0% (95% CI, 1.0–3.0%).

The present meta-analysis incorporated studies and extended follow-up data published after the search period covered by Yosofi et al. [5]. For fully oral SCRs, the pooled proportion of recurrent tuberculosis episodes in the primary GLMM analysis was 1.20% (95% CI, 0.70–2.03%). In the additional two-stage logit meta-analysis excluding zero-event cohorts, the corresponding estimate was 1.91% (95% CI, 1.24–2.96%), compared with 2.0% (95% CI, 1.0–3.0%) in the meta-analysis by Yosofi et al. [5].

### 3.5. Meta-Regression of Clinical Cohort Characteristics

In the univariable meta-regression analyses, no statistically significant associations were identified between the cohort-level proportions of patients with the examined characteristics and the frequency of recurrent tuberculosis episodes after SCRs (Table 13). For each 10-percentage-point increase in the proportion of patients with baseline sputum smear positivity, the OR was 1.06 (95% CI, 0.85–1.31; *p* = 0.607); for cavitary or destructive lung changes, 0.94 (95% CI, 0.74–1.20; *p* = 0.633); for HIV infection, 1.04 (95% CI, 0.83–1.31; *p* = 0.708); and for diabetes mellitus, 1.05 (95% CI, 0.53–2.07; *p* = 0.884). The 95% CIs included 1 in all models, and residual between-study heterogeneity remained (I^2^, 59.6–71.2%).

### 3.6. Small-Study Effects

Small-study effects were assessed in the primary single-arm dataset using a binomial-normal GLMM meta-regression with 1/√A as the predictor. No statistically significant association between study size and the frequency of recurrent tuberculosis episodes was identified (*p* = 0.597). In additional exploratory analyses restricted to the 20 cohorts with at least one event under the primary outcome definition, the sample-size-based random-effects regression test also showed no statistically significant evidence of small-study effects (*p* = 0.256), and Kendall’s rank-correlation test showed no statistically significant funnel-plot asymmetry (*p* = 0.113). In contrast, the SE-based Egger-type regression test indicated asymmetry (*p* = 0.0038). Given the inconsistency across diagnostic methods, the observed asymmetry was interpreted as an exploratory signal of small-study effects and not as evidence of publication bias.

### 3.7. Assessment of Certainty of Evidence According to GRADE

The certainty of evidence was rated as low for the pooled recurrence estimate after fully oral SCRs lasting ≤ 12 months, very low for the SCR subgroup estimates, and low for the comparative estimates of post-treatment recurrence (Table 14). Downgrading primarily reflected risk of bias, inconsistency across studies, and, for subgroup and comparative estimates, imprecision.

## 4. Discussion

In the present meta-analysis, post-treatment recurrence after SCRs was infrequent, with a pooled proportion of 1.24% in the primary analysis and 1.20% among fully oral regimens. These estimates, however, should not be interpreted as a single fixed recurrence risk because post-treatment follow-up varied considerably across studies and substantial between-study heterogeneity remained. Rather, they summarize outcomes across clinically diverse cohorts in which recurrence may have been influenced by baseline disease extent, mycobacterial burden, drug-resistance profile, regimen composition and effective drug activity, and the duration of subsequent observation.

The comparative analyses provide an additional perspective on these findings. In the full comparative set, neither the pooled RR nor the person-time-adjusted IRR showed a statistically significant difference between SCRs and comparator regimens. In the RCT subset, the RR was higher for SCRs, whereas the corresponding IRR, which accounted for differences in post-treatment follow-up duration, was not statistically significant. This divergence suggests that the apparent difference between treatment groups is sensitive to the analytical approach and particularly to how follow-up time after treatment completion is incorporated. Therefore, the higher RR observed in the RCT analysis cannot be attributed to shorter treatment duration alone.

### 4.1. Structural Lung Changes

The extent of structural lung changes, including destructive changes and bilateral pulmonary involvement, is considered an important clinical marker of increased relapse risk because it may reflect a high mycobacterial burden, persistence of viable mycobacteria in poorly vascularized lesions, and limited penetration of anti-tuberculosis drugs into cavities [31,33].

Meta-regression did not identify a statistically significant association between the cohort prevalence of cavitary or destructive lung changes and recurrence frequency. However, this does not exclude the clinical relevance of baseline disease extent and mycobacterial burden at the individual-patient level, as supported by individual studies. In the endTB-Q RCT [11], the authors indicated that baseline disease extent could be associated with relapse risk. Limited disease was defined as smear-negative status regardless of cavitation, whereas extensive disease was defined as sputum smear grade 2+ or 3+ regardless of cavitation, or grade 1+ in the presence of cavitation. The study authors reported that 8 of the 9 relapses in the SCR intervention group occurred among patients with extensive pulmonary disease.

### 4.2. Drug-Resistance Profile and Regimen Composition

The risk of recurrence after an SCR may depend not only on baseline disease severity but also on whether the prescribed regimen retained sufficient activity against the infecting M. tuberculosis strain.

In the comparative RCTs, BEAT TB [9] and endTB-Q [11] accounted for the largest numbers of recurrent episodes in the SCR groups.

In BEAT TB [9], the treatment regimen included bedaquiline, delamanid, linezolid, and levofloxacin and/or clofazimine. The regimen composition was modified after drug-susceptibility results became available. Levofloxacin was discontinued in the presence of fluoroquinolone resistance, whereas clofazimine was discontinued when the isolates were fluoroquinolone-susceptible. Recurrence was recorded in 10 patients in the SCR group, and bedaquiline resistance was detected at recurrence in two of them.

In endTB-Q [11], all patients had fluoroquinolone-resistant tuberculosis. The study evaluated a regimen containing bedaquiline, delamanid, linezolid, and clofazimine. Nine recurrent tuberculosis episodes were recorded in the SCR group, whereas no recurrent episodes occurred in the comparator group. Phenotypic and genotypic comparison of paired M. tuberculosis isolates identified previously unrecognized resistance to bedaquiline and clofazimine in five patients. One patient additionally had resistance to delamanid.

### 4.3. Contribution of Comorbid Conditions to Heterogeneity

HIV infection and diabetes mellitus may have contributed to the clinical heterogeneity of the included cohorts, although their contribution to between-study heterogeneity remained uncertain. Meta-regression did not identify a statistically significant association between the cohort prevalence of HIV infection or diabetes mellitus and the frequency of recurrence after SCRs.

Studies with high and low HIV prevalence showed differing numbers of recurrent episodes. In BEAT TB, a high proportion of patients with HIV infection was accompanied by a relatively large number of recurrent episodes after SCR treatment [9], whereas in endTB-Q a comparable number of recurrent episodes was recorded despite an almost complete absence of patients with HIV infection [11]. In Ndjeka et al., recurrence frequency remained low despite the highest proportion of patients with HIV infection [15]. The proportion of patients with HIV infection within a cohort did not necessarily reflect the severity of immunodeficiency and could not directly explain differences in recurrence frequency. The role of diabetes mellitus should likewise be interpreted cautiously because data were not reported in all studies and did not allow adjustment for glycemic control, disease duration, or the relationship between diabetes and the extent of tuberculosis.

### 4.4. Treatment Adherence

Treatment adherence and completion of the prescribed course may affect treatment effectiveness and the development of drug resistance [32]. Shortening the duration of therapy may reduce barriers to completing the full treatment course; however, the effect of adherence on the risk of recurrent tuberculosis was not directly evaluated in the present meta-analysis.

In TB-PRACTECAL [10], the SCR group had better treatment outcomes and a higher likelihood of completing the prescribed treatment course. Early treatment discontinuation was recorded in 37% (50/137) of patients in the comparator group and 8% (11/138) in the BPaLM SCR group. Loss to follow-up and premature withdrawal from the study occurred more frequently in the comparator group, indicating a substantially higher frequency of early treatment discontinuation with standard-of-care therapy in this study.

### 4.5. Strategy for Future Research

Future studies of long-term outcomes after SCRs should use the date of treatment completion as a common starting point for post-treatment follow-up. This would reduce between-group differences arising from unequal treatment durations.

To further evaluate risk factors for recurrent tuberculosis, future studies should prospectively record, at the individual-patient level, baseline mycobacterial burden, the presence and extent of cavitary or destructive lung changes, the drug-resistance profile, the activity of the prescribed regimen, HIV status and measures of immunological control, diabetes status and glycemic control, treatment interruptions, and completion of the prescribed treatment course.

Such individual-level data could be used to develop and externally validate prediction models for recurrent tuberculosis after treatment completion. The present aggregate-data meta-analysis cannot establish specific thresholds for individual characteristics or define validated categories of low, intermediate, and high individual risk. Given the clinical heterogeneity of the included studies, differences in post-treatment follow-up duration, and reliance on aggregate data, the certainty of evidence remained limited. According to GRADE, certainty was low for the main fully oral SCR estimate and very low for the subgroup estimates.

### 4.6. Limitations

The principal limitation of the analysis is the clinical and methodological heterogeneity of the included studies. Cohorts differed in treatment regimen composition, drug-resistance profiles, treatment duration, baseline disease severity, and duration of post-treatment follow-up. The search did not include Embase, Web of Science, Scopus, or CENTRAL; therefore, eligible studies indexed exclusively in these databases may have been missed.

The comparative analysis was limited by the small number of recurrent tuberculosis episodes, wide confidence intervals, and heterogeneity of the comparator groups. In several studies, follow-up was defined from randomization or study enrollment rather than from treatment completion, resulting in differences in the actual duration of post-treatment follow-up between the SCR and comparator groups.

When total person-time was not directly reported, it was estimated using the analytical denominator A and the available duration of post-treatment follow-up. In some studies, the duration of follow-up was reconstructed from the published overall follow-up horizon or used as an approximate group-level estimate. Therefore, the calculated person-years do not represent the sum of individual patient follow-up times.

The primary publications did not provide sufficient data to perform a time-to-event meta-analysis of tuberculosis recurrence or synthesize survival curves because individual event times, numbers of patients at risk at specific time points, and censoring data were reported incompletely and inconsistently.

Molecular comparison of baseline and recurrent M. tuberculosis isolates was unavailable in some publications; therefore, endogenous relapse could not always be distinguished from reinfection. The sensitivity analysis including all recurrent episodes reported by the original study authors increased the pooled estimate from 1.24% to 1.43%, indicating an effect of outcome definition, although the overall magnitude of the estimate remained low.

Meta-regression was based on aggregate cohort-level characteristics and therefore cannot assess individual-level risk factors. Formal methods for assessing small-study effects yielded inconsistent results: the primary GLMM small-study-effect test and the sample-size-based test were not statistically significant, and Kendall’s rank-correlation test showed no statistically significant funnel-plot asymmetry, whereas the SE-based Egger-type test indicated asymmetry. Therefore, publication bias can neither be confirmed nor reliably excluded.

## 5. Conclusions

1. In the primary single-arm GLMM analysis, the pooled proportion of recurrent tuberculosis episodes after SCRs lasting ≤ 12 months was 1.24% (95% CI, 0.79–1.93%), and after fully oral SCRs it was 1.20% (95% CI, 0.70–2.03%). The denominator sensitivity analysis using the original group population N yielded similar estimates.
2. For pretomanid-containing SCRs, the pooled proportion of recurrent tuberculosis episodes was 1.57% (95% CI, 0.85–2.85%); for SCRs lasting ≤ 39 weeks, 1.55% (95% CI, 0.93–2.56%); and for SCRs lasting ≤ 36 weeks, 1.95% (95% CI, 1.20–3.15%). These subgroups partially overlapped and did not represent independent comparisons of treatment duration.
3. Broadening the outcome definition to include all recurrent tuberculosis episodes reported by the original study authors increased the pooled proportion from 1.24% to 1.43% (95% CI, 0.95–2.16%) without materially changing the overall magnitude of the estimate.
4. In the full comparative analysis, no statistically significant difference was identified between SCRs and comparator groups: the RR was 1.35 (95% CI, 0.34–5.40), and the IRR accounting for estimated post-treatment person-time was 1.25 (95% CI, 0.50–3.15). In the analysis restricted to RCTs, the RR was 2.72 (95% CI, 1.25–5.89), whereas the IRR was 1.66 (95% CI, 0.81–3.43). The divergence between these estimates requires cautious interpretation of the comparative findings, taking differences in post-treatment follow-up duration into account.
5. Univariable meta-regression identified no statistically significant associations between the cohort-level proportions of patients with baseline sputum smear positivity, cavitary or destructive lung changes, HIV infection, or diabetes mellitus and the frequency of recurrent tuberculosis episodes. These findings do not exclude the individual prognostic relevance of these characteristics.

## Figures and Tables

**Table 1:** Key search concept blocks.

	Search Terms
Population	“Multidrug-resistant tuberculosis” OR “rifampicin-resistant tuberculosis” OR “MDR-TB” OR “RR-TB” OR “XDR-TB” OR “pre-XDR-TB” OR “drug-resistant tuberculosis”.
Intervention	“Short course chemotherapy” OR “shorter regimen” OR “short-course regimen” OR “all-oral regimen” OR “6-month regimen” OR “6-month treatment” OR “9-month regimen” OR “9-month treatment” OR “bedaquiline” OR “pretomanid” OR “BPaL” OR “BPaLM” OR “delamanid” OR “linezolid”.
Outcome	“relapse” OR “recurrence” OR “treatment outcome” OR “long-term outcome” OR “post-treatment outcome” OR “sustained cure” OR “durable response”
Design	“Randomized controlled trial” OR “clinical trial” OR “cohort study” OR “prospective study” OR “retrospective study”.

**Table 2:** Randomized clinical trials: RoB 2 assessment.

Study	D1	D2	D3	D4	D5	Overall
STREAM 1 (Nunn, 2019) [6]	L	L	SC	L	L	SC
STREAM Stage 2 (Goodall, 2024) [13]	L	L	L	L	L	L
MDR-END (Mok, 2022) [7]	L	L	SC	L	L	SC
NExT (Esmail, 2022) [12]	SC	H	SC	L	L	H
ZeNix (Conradie, 2022) [19]	L	L	SC	L	L	SC
TB-PRACTECAL (Nyang’wa, 2024) [10]	L	L	SC	SC	SC	SC
endTB (Guglielmetti, 2025) [8]	L	L	SC	L	L	SC
endTB-Q (Guglielmetti, 2025) [11]	L	L	SC	L	L	SC
BEAT TB (Conradie, 2026) [9]	L	L	SC	SC	SC	SC
Daniel et al., 2025 [27]	L	L	SC	SC	L	SC

RoB 2 domains: D1, randomization process; D2, deviations from intended interventions; D3, missing outcome data; D4, bias in measurement of the outcome; D5, selection of the reported result. Abbreviations: L, low risk; SC, some concerns; H, high risk.

**Table 3:** Non-randomized comparative studies: ROBINS-I assessment.

Study	D1	D2	D3	D4	D5	D6	D7	Overall
Ndjeka et al., 2022 [15] retrospective cohort; propensity score matching	M	M	L	M	L	L	L	M
Maryandyshev et al., 2019 [14] historical cohort comparison	L	L	L	L	L	L	L	L

ROBINS-I domains: D1, baseline comparability of the groups before treatment initiation; D2, patient selection procedures and the risk of forming non-comparable groups; D3, accuracy of classification of patients into the analyzed short-course regimen (SCR) or comparator regimen (CR); D4, deviations from the assigned regimen, including drug substitutions, premature discontinuation, treatment prolongation, or other treatment modifications; D5, completeness of outcome data and loss to follow-up; D6, methods used to ascertain and assess outcomes, including post-treatment recurrence; D7, completeness of results reporting. Abbreviations: L, low risk; M, moderate risk.

**Table 4:** Single-arm cohort studies: adapted Newcastle-Ottawa Scale assessment.

Study	Cohort	Regimen Data	Post-Treatment Recurrence	Outcome Assessment	Follow-Up Period	Follow-Up Completeness
Nix-TB (Conradie, 2020) [16]	+	+	+	+	+	±
BIG cohort (Haley, 2023) [25]	±	+	+	±	+	−
Burhan et al., 2025 [24]	±	+	+	+	+	−
Fardhdiani et al., 2025 [21]	+	+	+	±	+	−
Korotych et al., 2024 [17]	±	+	+	+	+	−
Le T.N. Anh et al., 2020 [29]	±	+	+	−	+	−
Schwœbel et al., 2020 [18]	+	+	+	+	+	−
Sinha et al., 2025 [22]	+	+	+	+	+	−
BEAT-India (Padmapriyadarsini, 2023) [23]	+	+	+	+	+	±
Nguyen et al., 2026 [28]	+	+	+	±	+	−
Golubchikov et al., 2023 [30]	+	+	+	+	+	+
Khimova et al., 2025 [20]	±	+	+	±	+	+
TRUST-plus (Li et al., 2025) [26]	+	+	+	+	+	±

Note. +, criterion fully met; ±, criterion partially met; −, criterion not met.

**Table 5:** Single-arm meta-analysis.

Analysis	Number of Cohorts [Source]	r/A	Method
Primary GLMM analysis: all SCRs	26 [6,7,8,9,10,11,12,13] (9 months), [13] (6 months), [14,15,16,17,18,19,20,21,22,23,24,25,26,27,28,29,30]	98/6954	Primary analysis, GLMM
Fully oral SCRs	20 [7,8,9,10,11,12,13] (9 months), [15,16,17,19,20,21,22,23,24,25,26,27,28]	74/5532	Sensitivity analysis, GLMM
Pretomanid-containing SCRs	7 [10,16,19,22,24,25,27]	21/1260	Subgroup analysis, GLMM
SCRs ≤ 39 weeks	16 [8,9,10,11,12,13] (6 months), [16,17,19,20,22,23,24,25,26,27]	70/4594	Subgroup analysis, GLMM
SCRs ≤ 36 weeks	11 [9,10,12,13] (6 months), [16,19,22,23,24,25,27]	37/1747	Subgroup analysis, GLMM

Note. r, number of post-treatment recurrent tuberculosis episodes used in the primary analysis. A, number of patients in whom recurrent tuberculosis was assessed after treatment completion; GLMM, generalized linear mixed model.

**Table 6:** Comparative meta-analysis.

Analysis	Number of Studies [Source]	SCR r/A	Comparator r/A
Primary comparative analysis: all comparative studies	9 [6,7,8,9,10,11,12,14,15]	30/1736	15/1176
RCTs	7 [6,7,8,9,10,11,12]	29/1084	5/626

Note. r, number of recurrent tuberculosis episodes; A, post-treatment analytical denominator; SCR, short-course regimen; RCT, randomized controlled trial.

**Table 7:** Characteristics of the Included Studies.

Study	Country(ies)	DR Profile	Group	Regimen	r/N	r/A	Treatment, Weeks	Post-Treatment Follow-Up, Weeks
STREAM 1; Nunn A.J. et al., 2019 [6,43]	Ethiopia; Mongolia; South Africa; Viet Nam	MDR-TB	SCR	Mfx Cfz E Z Km Hh Pto	7/253	7/212	40	91.9
			CR	2011 WHO regimen	0/130	0/109	82.7	49.3
MDR-END; Mok J. et al., 2022 [7]	South Korea	MDR-TB	SCR	Dlm Lfx Lzd Z	1/72	1/61	40	64
			CR	Individualized regimen	0/85	0/64	87	17
endTB; Guglielmetti L. et al., 2025 [8]	Georgia; India; Kazakhstan; Lesotho; Pakistan; Peru; South Africa	MDR-TB	SCR	9BLMZ: Bdq Lzd Mfx Z; 9BCLLfxZ: Bdq Cfz Lzd Lfx Z; 9BDLLfxZ: Bdq Dlm Lzd Lfx Z	0/355	0/314	39	65
			CR	Individualized regimen	0/119	0/92	78.1	25.9
BEAT TB; Conradie F. et al., 2026 [9]	South Africa	RR-TB, including FQ-resistant TB	SCR	Bdq Dlm Lzd Lfx and/or Cfz	10/202	10/188	24	52
			CR	9-month South African standard regimen; individualized regimen for FQ resistance	4/200	4/178	NA	37
TB-PRACTECAL; Nyang’wa B.-T. et al., 2024 [10]	Belarus; Uzbekistan; South Africa	MDR-TB	SCR	Bdq Pa Lzd Mfx	1/138	1/126	24	84
			CR	Individualized regimen	1/137	1/81	NA	50
endTB-Q; Guglielmetti L. et al., 2025 [11]	India; Kazakhstan; Lesotho; Pakistan; Peru; Viet Nam	pre-XDR-TB	SCR	Bdq Dlm Lzd Cfz	9/163	9/150	39	63.1
			CR	Individualized regimen	0/84	0/72	78	23.9
NExT; Esmail A. et al., 2022 [12]	South Africa	MDR-TB	SCR	Lzd Bdq Lfx Z Trd Eto Hh	1/49	1/33	26	52
			CR	Individualized regimen	0/44	0/30	NA	52
STREAM Stage 2, 9 months; Goodall R.L. et al., 2024 [13]	Ethiopia; Georgia; India; Moldova; Mongolia; South Africa; Uganda	MDR-TB	SCR	Bdq Lfx Cfz E Z Hh Pto	3/196	3/168	40	92
			CR	9-month injectable-containing regimen	1/187	1/142	40	92
STREAM Stage 2, 6 months; Goodall R.L. et al., 2024 [13]	Ethiopia; Georgia; India; Moldova; Mongolia; South Africa; Uganda	MDR-TB	SCR	Bdq Lfx Cfz Z Hh Km	2/134	2/127	28	104
			CR	9-month injectable-containing regimen	1/127	1/94	40	92
Maryandyshev A.O. et al., 2019 [14]	Russian Federation	MDR-TB	SCR	Km/Cm Lfx/Mfx Z Pto Cs/E	0/145	0/145	53.3	NA
			CR	Individualized regimen	6/129	6/129	75.4	NA
Ndjeka N. et al., 2022 [15]	South Africa	MDR-TB	SCR	Bdq Lfx/Mfx Cfz E Z Hh Eto/Pto	1/507	1/507	52	58.5
			CR	Km Lfx/Mfx Cfz E Z Hh Eto/Pto	4/421	4/421	NA	58.5
Nix-TB; Conradie F. et al., 2020 [16]	South Africa	MDR/XDR-TB	SCR	Bdq Pa Lzd	2/109	2/102	26	26
Korotych O. et al., 2024 [17]	13 countries of the WHO European Region	MDR-TB	SCR	Bdq Lzd Lfx Cfz Cs (2532); Bdq Lzd Lfx Cfz Dlm (75); Lzd Lfx Cfz Dlm (29)	22/2181	22/2181	39	52
Schwœbel et al., 2020 [18]	Benin; Burkina Faso; Burundi; Cameroon; Central African Republic; Côte d’Ivoire; Democratic Republic of the Congo; Niger; Rwanda	MDR-TB	SCR	Km Mfx Cfz E Z Hh Pto → Mfx Cfz E Z	14/823	14/823	47.7	NA
ZeNix; Conradie F. et al., 2022 [19]	South Africa; Russia; Georgia; Moldova	MDR/pre-XDR-TB	SCR	Bdq Pa Lzd	6/176	6/160	26	NA
Khimova E.S. et al., 2025 [20]	Russian Federation	MDR/pre-XDR-TB	SCR	Lfx Bdq Lzd Cfz (134); Lfx Bdq Dlm Cfz (1); Bdq Lzd Dlm Cfz (32)	2/137	2/137	39	52
Fardhdiani V. et al., 2025 [21]	Ukraine	MDR/pre-XDR-TB	SCR	Bdq Lzd Lfx/Dlm Cfz Cs	1/234	1/234	52	52
Sinha A. et al., 2025 [22]	Belarus; Uzbekistan	MDR/pre-XDR-TB	SCR	Bdq Pa Lzd Mfx; Bdq Pa Lzd Cfz	2/383	2/383	24	52
BEAT-India; Padmapriyadarsini C. et al., 2023 [23]	India	MDR/pre-XDR-TB	SCR	Bdq Dlm Lzd Cfz	3/153	3/139	36	NA
Burhan E. et al., 2025 [24]	Indonesia	MDR/pre-XDR-TB	SCR	Bdq Pa Lzd	0/69	0/69	26	26
BIG cohort; Haley C.A. et al., 2023 [25]	USA	RR-TB or rifampin-intolerant TB	SCR	Bdq Pa Lzd	2/68	2/68	26	NA
TRUST-plus; Li Y. et al., 2025 [26]	China	pre-XDR-TB	SCR	Bdq Lzd Cs Z/Cfz	0/80	0/65	39	48.4
Daniel B.D. et al., 2025 [27]	India	pre-XDR-TB	SCR	Bdq Pa Lzd	8/378	8/352	26	48
Nguyen T.M.P. et al., 2026 [28]	Viet Nam	MDR-TB	SCR	Bdq Cfz Lzd Lfx Z	0/95	0/95	47.7	156
Le T.N. Anh et al., 2020 [29]	Viet Nam	MDR-TB	SCR	Lfx Km Cfz Pto E Hh Z	0/79	0/79	47.7	52
Golubchikov P.N. et al., 2023 [30]	Russian Federation	MDR/pre-XDR-TB	SCR	Bdq Lzd FQ with individualized companion drugs; Km/Am/Cm in 81%	1/36	1/36	43.33	NA

Note. r, number of post-treatment recurrent tuberculosis episodes used in the primary analysis; N, group size reported in the publication; A, number of patients in whom recurrent tuberculosis was assessed after successful treatment completion. Bdq, bedaquiline; Pa, pretomanid; Dlm, delamanid; Lzd, linezolid; Mfx, moxifloxacin; Lfx, levofloxacin; Cfz, clofazimine; Cs, cycloserine; E, ethambutol; Z, pyrazinamide; Km, kanamycin; Cm, capreomycin; Am, amikacin; Pto, prothionamide; Hh, high-dose isoniazid; Trd, terizidone; Eto, ethionamide; FQ, fluoroquinolone; SCR, short-course regimen; CR, comparator regimen; NA, not available. Regimen duration represents the nominal planned duration reported by the source or its descriptive conversion to weeks.

**Table 8:** Clinical Characteristics of the Included Cohorts.

Study	Baseline Sputum Smear Positive, %	Cavitary or Destructive Lung Changes, %	HIV, %	Diabetes Mellitus, %
STREAM 1 [6,43]	96.8 (245/253)	77.0 (184/239)	33.6 (85/253)	8.3 (21/252)
MDR-END [7]	30.4 (24/79)	48.1 (38/79)	0.0 (0/79)	20.3 (16/79)
endTB trial [8]	80.3 (285/355)	59.2 (210/355)	13.0 (46/355)	16.3 (58/355)
BEAT TB [9]	43.3 (88/203)	18.2 (37/203)	51.7 (105/203)	NA
TB-PRACTECAL [10]	62.3 (86/138)	55.1 (76/138)	24.6 (34/138)	NA
endTB-Q [11]	72.4 (118/163)	66.3 (108/163)	0.6 (1/163)	22.7 (37/163)
NExT trial [12]	61.2 (30/49)	53.1 (26/49)	55.1 (27/49)	NA
STREAM Stage 2 (6 months) [13]	97.8 (131/134)	75.0 (93/124)	15.7 (21/134)	9.8 (13/132)
STREAM Stage 2 (9 months) [13]	99.0 (194/196)	75.5 (139/184)	13.8 (27/196)	8.2 (16/195)
Maryandyshev A.O. [14]	57.7 (105/182)	NA	6.6 (12/182)	NA
Ndjeka N. [15]	49.2 (297/604)	NA	71.7 (493/688)	NA
Nix-TB [16]	NA	84.4 (92/109)	51.4 (56/109)	NA
Korotych O. [17]	58.3 (1519/2604)	57.7 (1518/2631)	10.3 (270/2633)	8.3 (220/2635)
Schwœbel V. [18]	NA	NA	19.9 (200/1006)	NA
ZeNix [19]	50.3 (91/181)	61.9 (112/181)	19.9 (36/181)	5.0 (9/181)
Khimova E.S. [20]	74.3 (124/167)	68.3 (114/167)	13.2 (22/167)	8.4 (14/167)
Fardhdiani V. [21]	NA	NA	23.0 (69/300)	4.3 (13/300)
Sinha A. [22]	33.4 (226/677)	47.8 (319/667)	5.3 (36/677)	8.9 (60/677)
BEAT-India [23]	95.2 (157/165)	50.9 (84/165)	NA	11.5 (19/165)
Burhan E. [24]	NA	34.5 (29/84)	1.2 (1/84)	36.9 (31/84)
BIG cohort; Haley 2023 [25]	54.0 (34/63)	46.0 (29/63)	5.7 (4/70)	20.0 (14/70)
TRUST-plus; Li 2025 [26]	97.5 (78/80)	82.5 (66/80)	0.0 (0/80)	8.8 (7/80)
Daniel B.D. [27]	92.3 (372/403)	48.6 (193/397)	0.5 (2/403)	12.2 (49/403)
Nguyen T.M.P. [28]	51.5 (53/103)	NA	1.2 (1/84)	NA
Le T.N. Anh [29]	NA	NA	NA	NA
Golubchikov P.N. [30]	57.1 (24/42)	69.0 (29/42)	26.2 (11/42)	NA

Note. HIV, human immunodeficiency virus; NA, data not reported in the publication. Data are presented for the short-course regimen groups. Denominators shown in parentheses correspond to the number of patients for whom the specific clinical characteristic was reported in the primary source and may therefore differ from N and A used in the meta-analysis.

**Table 9:** Numbers of Recurrent Tuberculosis Episodes and Patients by Analysis Set.

Analysis	Number of Cohorts [References]	r/A	r/N
All SCRs ≤ 12 months	26 [6,7,8,9,10,11,12,13,14,15,16,17,18,19,20,21,22,23,24,25,26,27,28,29,30]	98/6954	98/7215
Fully oral SCRs	20 [7,8,9,10,11,12,13] (9 months), [15,16,17,19,20,21,22,23,24,25,26,27,28]	74/5532	74/5745
Pretomanid-containing SCRs	7 [10,16,19,22,24,25,27]	21/1260	21/1321
SCRs ≤ 39 weeks	16 [8,9,10,11,12,13] (6 months), [16,17,19,20,22,23,24,25,26,27]	70/4594	70/4775
SCRs ≤ 36 weeks	11 [9,10,12,13] (6 months), [16,19,22,23,24,25,27]	37/1747	37/1859

Note. SCR, short-course regimen; r, number of recurrent tuberculosis episodes; A, number of patients in whom a recurrent tuberculosis episode could be assessed after treatment completion; N, size of the corresponding group reported in the publication. The r/A and r/N ratios are descriptive and do not represent pooled meta-analytic estimates.

**Table 10:** Results of the Single-Arm GLMM Analyses of the Proportion of Recurrent Tuberculosis Episodes.

Analysis	Number of Cohorts	Calculation	r/n	Proportion, %	95% CI, %	95% Prediction Interval, %	τ^2^	I^2^, %	*p* for Heterogeneity
All SCRs ≤ 12 months	26	r/A	98/6954	1.24	0.79–1.93	0.24–6.21	0.6679	70.2	<0.001
All SCRs ≤ 12 months	26	r/N	98/7215	1.17	0.76–1.81	0.24–5.53	0.6114	68.4	<0.001
Fully oral SCRs	20	r/A	74/5532	1.20	0.70–2.03	0.20–6.95	0.7859	72.5	<0.001
Fully oral SCRs	20	r/N	74/5745	1.13	0.67–1.89	0.19–6.24	0.7396	71.3	<0.001
Pretomanid-containing SCRs	7	r/A	21/1260	1.57	0.85–2.85	0.53–4.53	0.2131	36.0	0.070
Pretomanid-containing SCRs	7	r/N	21/1321	1.50	0.84–2.68	0.56–4.00	0.1720	31.3	0.092
SCRs ≤ 39 weeks	16	r/A	70/4594	1.55	0.93–2.56	0.33–7.04	0.5744	69.2	<0.001
SCRs ≤ 39 weeks	16	r/N	70/4775	1.45	0.88–2.38	0.31–6.41	0.5506	68.3	<0.001
SCRs ≤ 36 weeks	11	r/A	37/1747	1.95	1.20–3.15	0.66–5.64	0.2529	43.6	0.030
SCRs ≤ 36 weeks	11	r/N	37/1859	1.83	1.14–2.93	0.65–5.04	0.2254	40.8	0.044

Note. GLMM, binomial-normal random-effects generalized linear mixed model with a logit link; r, number of recurrent tuberculosis episodes; n, denominator corresponding to the specified calculation (A or N); CI, confidence interval; 95% prediction interval, expected range of the true proportion of recurrent tuberculosis episodes in a new comparable cohort after accounting for between-study variability; τ^2^, between-study variance on the logit scale; I^2^, measure of between-study heterogeneity; *p*, *p* value for the test of between-study heterogeneity.

**Table 11:** Sensitivity Analysis: Two-Stage Logit Meta-Analysis Excluding Zero-Event Cohorts.

Analysis	Cohorts in Model/Total	Calculation	r/n	Proportion, %	95% CI, %
All SCRs ≤12 months	20/26	r/A	98/6187	2.02	1.42–2.85
All SCRs ≤12 months	20/26	r/N	98/6392	1.89	1.35–2.65
Fully oral SCRs	16/20	r/A	74/4989	1.91	1.24–2.96
Fully oral SCRs	16/20	r/N	74/5146	1.79	1.17–2.74
Pretomanid-containing SCRs	6/7	r/A	21/1191	2.03	1.18–3.49
Pretomanid-containing SCRs	6/7	r/N	21/1252	1.95	1.16–3.24
SCRs ≤ 39 weeks	13/16	r/A	70/4146	2.25	1.45–3.46
SCRs ≤ 39 weeks	13/16	r/N	70/4271	2.11	1.38–3.21
SCRs ≤ 36 weeks	10/11	r/A	37/1678	2.42	1.56–3.75
SCRs ≤ 36 weeks	10/11	r/N	37/1790	2.25	1.45–3.46

Note. Random-effects inverse-variance model. Zero-event cohorts were excluded without applying a continuity correction.

**Table 12:** Comparative Meta-Analysis of SCRs and Comparator Groups.

Analysis	Total/Estimable	Effect Measure	SCR	Comparator Group	Estimate (95% CI)	I^2^, %
All studies	9/8 [6,7,8,9,10,11,12,14,15]	RR, r/A	30/1736	15/1176	1.35 (0.34–5.40)	41.2
All studies	9/8 [6,7,8,9,10,11,12,14,15]	RR, r/N	30/1884	15/1349	1.45 (0.36–5.83)	41.4
Studies with available person-time	8/7 [6,7,8,9,10,11,12,15]	IRR, r/PY	30/2019.18	9/911.34	1.25 (0.50–3.15)	0.0
RCTs	7/6 [6,7,8,9,10,11,12]	RR, r/A	29/1084	5/626	2.72 (1.25–5.89)	0.0
RCTs	7/6 [6,7,8,9,10,11,12]	RR, r/N	29/1232	5/799	2.95 (1.49–5.86)	0.0
RCTs with available person-time	7/6 [6,7,8,9,10,11,12]	IRR, r/PY	29/1448.80	5/437.72	1.66 (0.81–3.43)	0.0

Note. SCR, short-course regimen; r, number of recurrent tuberculosis episodes; A, post-treatment analytical denominator; N, original size of the corresponding analyzed group; PY, estimated post-treatment follow-up time in person-years; RR, risk ratio; IRR, incidence rate ratio; I^2^, measure of between-study heterogeneity. For the RR analyses in the full comparative set and the RCT analysis, endTB [8], in which no recurrent tuberculosis episodes occurred in either group, did not provide an estimable relative effect; therefore, the pooled RRs were calculated from 8 of 9 comparisons and 6 of 7 RCTs, respectively. In the IRR analysis, an estimable effect was obtained for 7 of the 8 studies with available person-time.

**Table 13:** Meta-regression of clinical cohort characteristics.

Covariate	Number of Cohorts	β per +10 pp	OR (95% CI)	*p*
Cavitary or destructive lung changes	20	−0.059	0.94 (0.74–1.20)	0.633
HIV infection	24	0.044	1.04 (0.83–1.31)	0.708
Diabetes mellitus	16	0.050	1.05 (0.53–2.07)	0.884
Baseline sputum smear positivity	21	0.057	1.06 (0.85–1.31)	0.607

Note. β, meta-regression coefficient per 10-percentage-point increase in the covariate; OR, odds ratio for a recurrent tuberculosis episode per 10-percentage-point increase in the covariate; CI, confidence interval; *p*, *p* value; pp, percentage points.

**Table 14:** Assessment of certainty of evidence according to GRADE.

Outcome Assessed	Risk of Bias	Inconsistency	Indirectness	Imprecision	Publication Bias	Certainty
Fully oral SCRs ≤12 months	serious	serious	not serious	not serious	cannot be excluded	low
SCR subgroups	serious	serious	not serious	serious	cannot be excluded	very low
Comparison of post-treatment recurrence after SCRs and comparator regimens	serious	not serious	not serious	serious	cannot be excluded	low

## Data Availability

The study-level dataset and analytical calculations are provided in Supplementary File S3. The review protocol is registered in OSF Registries (DOI: http://doi.org/10.17605/OSF.IO/4NUK5). Statistical code and the screening script are available from the corresponding author upon reasonable request.

## Supplementary Materials

The following supporting information can be downloaded at: https://www.mdpi.com/article/10.3390/jcm15218273/s1, Supplementary File S1: PRISMA 2020 checklist; Supplementary File S2: Full Search Strategy and Study-Selection Procedures; Supplementary File S3: extracted study-level dataset and analytical calculations.

## Abbreviations

DR-TB, drug-resistant tuberculosis; RR-TB, rifampicin-resistant tuberculosis; MDR-TB, multidrug-resistant tuberculosis; pre-XDR-TB, pre-extensively drug-resistant tuberculosis; XDR-TB, extensively drug-resistant tuberculosis; SCR, short-course regimen; CR, comparator regimen; RCT, randomized controlled trial; GLMM, generalized linear mixed model; REML, restricted maximum likelihood; CI, confidence interval; RR, risk ratio; OR, odds ratio; IR, incidence rate; IRR, incidence rate ratio; PY, person-years; WHO, World Health Organization; OSF, Open Science Framework; PRISMA, Preferred Reporting Items for Systematic Reviews and Meta-Analyses; GRADE, Grading of Recommendations Assessment, Development and Evaluation; RoB 2, Risk of Bias 2; ROBINS-I, Risk Of Bias In Non-randomized Studies of Interventions; HIV, human immunodeficiency virus.
