# Supplementary material for: Frequency of Post-Treatment Recurrence After Short-Course Regimens for Drug-Resistant Tuberculosis: A Systematic Review and Meta-Analysis
Source: J Clin Med. 2026 Oct 23;15(21):8273. doi: 10.3390/jcm15218273 (PMC13764048; doi:10.3390/jcm15218273)
Supplement: Supplementary file 1 [file jcm-15-08273-s001.zip › jcm-4524953-Supplementary File S2.pdf]

Supplementary File S2

Full Search Strategy and Study-Selection Procedures

Frequency of Post-Treatment Recurrence After Short-Course Regimens for Drug-Resistant Tuberculosis: A Systematic Review and Meta-Analysis

Protocol registration

The systematic review and meta-analysis protocol was registered prospectively in OSF Registries (DOI: 10.17605/OSF.IO/4NUK5).

Information sources

PubMed and Europe PMC were searched as the principal bibliographic sources. ClinicalTrials.gov and the Chinese Clinical Trial Registry (ChiCTR) were searched as supplementary registry sources. Reference lists of included publications and previous reviews of short-course treatment regimens for drug-resistant tuberculosis were also screened. Russian-language evidence was searched in eLIBRARY.RU and the electronic archives of relevant Russian journals, including Tuberculosis and Lung Diseases («Туберкулёз и болезни лёгких»). Embase, Web of Science, Scopus, and CENTRAL were not searched.

Search period and eligibility limits

The search covered publications from 1 January 2018 to 1 May 2026; the principal electronic search was last run on 1 May 2026. Reports published in English, Russian, or Chinese were eligible. Eligible study designs included randomized controlled trials, clinical trials, prospective and retrospective cohort studies, and other cohort studies reporting post-treatment follow-up and tuberculosis relapse or recurrence.

Search yield

Source	Records identified	Search end date
PubMed	99	1 May 2026
Europe PMC	139	1 May 2026
ClinicalTrials.gov	95	1 May 2026
ChiCTR	399	1 May 2026
Total	732	

Search concept blocks

Concept	Keywords
Population	"Multidrug-resistant tuberculosis" OR "rifampicin-resistant tuberculosis" OR "MDR-TB" OR "RR-TB" OR "XDR-TB" OR "pre-XDR-TB" OR "drug-resistant tuberculosis"
Intervention	"short course chemotherapy" OR "shorter regimen" OR "short-course regimen" OR "all-oral regimen" OR "6-month regimen" OR "6-month treatment" OR "9-month regimen" OR "9-month treatment" OR "bedaquiline" OR "pretomanid" OR "BPaL" OR "BPaLM" OR "delamanid" OR "linezolid"
Outcome	"relapse" OR "recurrence" OR "treatment outcome" OR "long-term outcome" OR "post-treatment outcome" OR "sustained cure" OR "durable response"
Study design	"randomized controlled trial" OR "clinical trial" OR "cohort study" OR "prospective study" OR "retrospective study"

Common Boolean search string used in PubMed and Europe PMC

("Multidrug-resistant tuberculosis" OR "rifampicin-resistant tuberculosis" OR "MDR-TB" OR "RR-TB" OR "XDR-TB" OR "pre-XDR-TB" OR "drug-resistant tuberculosis")

AND ("short course chemotherapy" OR "shorter regimen" OR "all-oral regimen" OR "6-month regimen" OR "6-month treatment" OR "9-month regimen" OR "9-month treatment" OR "bedaquiline" OR "pretomanid" OR "BPaL" OR "BPaLM" OR "delamanid" OR "linezolid")
AND ("relapse" OR "recurrence" OR "treatment outcome" OR "long-term outcome" OR "post-treatment outcome" OR "sustained cure" OR "durable response")
AND ("randomized controlled trial" OR "clinical trial" OR "cohort study" OR "prospective study" OR "retrospective study")

The query was entered without additional field restrictions; database-native query processing was used. Publication-date and language limits were applied separately.

PubMed

The common Boolean search string shown above was entered in PubMed without additional field tags. PubMed automatic term mapping was retained. The publication-date limit was 1 January 2018 to 1 May 2026, and eligible languages were English, Russian, and Chinese. The search identified 99 records.

Europe PMC

The same common Boolean search string was entered in Europe PMC without restricting terms to TITLE_ABS fields. Europe PMC native query processing was retained. The publication-date limit was 1 January 2018 to 1 May 2026, and eligible languages were English, Russian, and Chinese. The search identified 139 records.

ClinicalTrials.gov

The registry was searched for completed interventional or observational records concerning drug-resistant tuberculosis, shorter treatment regimens, and post-treatment relapse or recurrence. The registry search combined the following concepts:

Condition/disease: "drug-resistant tuberculosis" OR "multidrug-resistant tuberculosis" OR "MDR-TB" OR "RR-TB" OR "pre-XDR-TB" OR "XDR-TB"
Other terms: "shorter regimen" OR "short-course" OR "short course" OR "6-month" OR "9-month" OR "all-oral" OR "bedaquiline" OR "pretomanid" OR "BPaL" OR "BPaLM" OR "delamanid" OR "linezolid"
Outcome concepts: "relapse" OR "recurrence" OR "post-treatment" OR "long-term outcome" OR "sustained cure" OR "durable response"

Search period: 1 January 2018 to 1 May 2026. Records identified: 95.

Chinese Clinical Trial Registry (ChiCTR)

The registry was searched using terms for drug-resistant tuberculosis, shorter treatment, and post-treatment relapse or recurrence in the available registry fields:

("drug-resistant tuberculosis" OR "multidrug-resistant tuberculosis" OR "MDR-TB" OR "RR-TB" OR "pre-XDR-TB" OR "XDR-TB")
AND ("shorter regimen" OR "short-course" OR "short course" OR "6-month" OR "9-month" OR "bedaquiline" OR "pretomanid" OR "BPaL" OR "BPaLM" OR "delamanid" OR "linezolid")
AND ("relapse" OR "recurrence" OR "post-treatment outcome" OR "long-term outcome" OR "sustained cure" OR "durable response")

Search period: 1 January 2018 to 1 May 2026. Records identified: 399.

Russian-language search

A supplementary Russian-language search was conducted in eLIBRARY.RU and the electronic archives of relevant Russian journals using Russian-language equivalents of the same population, intervention, and outcome concepts. Three eligible Russian-language reports were included in the review [14, 20, 30]. The same eligibility criteria were applied irrespective of publication language.

Reference-list searching

Reference lists of all included publications and previous systematic reviews of short-course treatment regimens for drug-resistant tuberculosis were screened for additional eligible reports. The same eligibility criteria were applied to all reports.

Targeted pre-submission update

A targeted pre-submission update was performed for studies already known from trial registries, conference reports, or included sources. The final peer-reviewed publication of the BEAT Tuberculosis trial replaced the previously available report of the same study; the number of included studies did not change.

Deduplication and study selection

Search results were combined in a single screening dataset. Duplicate records were removed before screening. Two reviewers independently screened records and assessed full-text eligibility. A Python script supported deduplication and screening. Final eligibility decisions were made manually by the reviewers.

Stage	Number
Records identified in the four electronic sources	732
Duplicate records removed	84
Records screened	648
Records excluded before full-text assessment	457
Full-text reports assessed for eligibility	191
Full-text reports excluded	166
Studies included in the systematic review	25
Analytical short-course regimen cohorts	26

Reasons for full-text exclusion

Reason	Number
No eligible relapse or recurrence data	104
Insufficient post-treatment follow-up information and/or population at risk not identifiable	43
Eligible group size <20, including Goswami et al. (2022)	19
Total	166
