# Supplementary material for: Frequency of Post-Treatment Recurrence After Short-Course Regimens for Drug-Resistant Tuberculosis: A Systematic Review and Meta-Analysis
Source: J Clin Med. 2026 Oct 23;15(21):8273. doi: 10.3390/jcm15218273 (PMC13764048; doi:10.3390/jcm15218273)
Supplement: Supplementary file 1 [file jcm-15-08273-s001.zip › jcm-4524953-Supplementary File S1 PRISMA Checklist.pdf]

Supplementary File S1

PRISMA 2020 checklist

Frequency of Post-Treatment Recurrence After Short-Course Regimens for Drug-Resistant Tuberculosis: A Systematic Review and Meta-Analysis

Section/topic	Item #	Checklist item	Location where item is reported
**Title**	1	Identify the report as a systematic review.	Title page
**Abstract**	2	See the PRISMA 2020 for Abstracts checklist.	Abstract
**Introduction**	3	Describe the rationale for the review in the context of existing knowledge.	Introduction
**Introduction**	4	Provide an explicit statement of the objective(s) or question(s) the review addresses.	Objective; Study Objectives
**Methods**	5	Specify the inclusion and exclusion criteria for the review and how studies were grouped for the syntheses.	Inclusion Criteria; Exclusion Criteria; Selection of Groups for Meta-Analysis; Statistical Analysis
**Methods**	6	Specify all databases, registers, websites, organisations, reference lists, and other sources searched or consulted, with the date when each source was last searched.	Materials and Methods; Study Selection Process; Supplementary File S2
**Methods**	7	Present the full search strategies for all databases, registers, and websites, including any filters and limits used.	Materials and Methods; Table 1; Supplementary File S2
**Methods**	8	Specify the methods used to decide whether a study met the inclusion criteria, including how many reviewers screened records and reports, whether they worked independently, and any automation tools used.	Study Selection Process; Data Extraction
**Methods**	9	Specify the methods used to collect data from reports, including how many reviewers collected data and whether they worked independently, and any processes for obtaining or confirming data.	Data Extraction
**Methods**	10a	List and define all outcomes for which data were sought. Specify whether all results compatible with each outcome domain in each study were sought.	Data Extraction; Statistical Analysis
**Methods**	10b	List and define all other variables for which data were sought.	Data Extraction; Tables 7-8
**Methods**	11	Specify the methods used to assess risk of bias in the included studies, including details of the tools used.	Risk-of-Bias Assessment of Included Studies; Tables 2-4
**Methods**	12	Specify for each outcome the effect measure(s) used in the synthesis or presentation of results.	Statistical Analysis; Tables 5-6
**Methods**	13a	Describe the processes used to decide which studies were eligible for each synthesis.	Selection of Groups for Meta-Analysis; Statistical Analysis; Tables 5-6
**Methods**	13b	Describe any methods required to prepare the data for presentation or synthesis, such as handling missing summary statistics or data conversions.	Data Extraction; Statistical Analysis; Supplementary File S3
**Methods**	13c	Describe any methods used to tabulate or visually display results of individual studies and syntheses.	Figure 1; Tables 2-14; Supplementary File S3
**Methods**	13d	Describe any methods used to synthesize results and provide rationale for the chosen methods.	Statistical Analysis; Tables 5-6; Supplementary File S3
**Methods**	13e	Describe any methods used to explore possible causes of heterogeneity among study results.	Statistical Analysis; Table 13
**Methods**	13f	Describe any sensitivity analyses conducted to assess robustness of the synthesized results.	Statistical Analysis; Results; Supplementary File S3
**Methods**	14	Describe any methods used to assess risk of bias due to missing results in a synthesis.	Statistical Analysis; Results; Supplementary File S3
**Methods**	15	Describe any methods used to assess certainty or confidence in the body of evidence for an outcome.	Risk-of-Bias Assessment of Included Studies; GRADE assessment; Table 14
**Results**	16a	Describe the results of the search and selection process, from number of records identified to number of studies included, ideally using a flow diagram.	Study Selection Process; Figure 1; Results
**Results**	16b	Cite studies that might appear to meet the inclusion criteria but were excluded, and explain why they were excluded.	Figure 1; Supplementary File S2
**Results**	17	Cite each included study and present its characteristics.	Results; Tables 7-8
**Results**	18	Present assessments of risk of bias for each included study.	Tables 2-4
**Results**	19	For all outcomes, present, for each study, summary statistics for each group and, where applicable, effect estimates and precision.	Results; Tables 7-12; Supplementary File S3
**Results**	20a	For each synthesis, briefly summarize the characteristics and risk of bias among contributing studies.	Results; Tables 2-12
**Results**	20b	Present results of all statistical syntheses conducted, including summary estimates, precision, and heterogeneity.	Results; Tables 9-12; Supplementary File S3
**Results**	20c	Present results of investigations of possible causes of heterogeneity among study results.	Results; Table 13

**Results**	20d	Present results of all sensitivity analyses conducted to assess robustness of synthesized results.	Results; Statistical Analysis; Supplementary File S3
**Results**	21	Present assessments of risk of bias due to missing results for each synthesis assessed.	Results; small-study-effects assessment; Supplementary File S3
**Results**	22	Present assessments of certainty or confidence in the body of evidence for each outcome assessed.	Results; Table 14
**Discussion**	23a	Provide a general interpretation of the results in the context of other evidence.	Discussion
**Discussion**	23b	Discuss any limitations of the evidence included in the review.	Discussion; Limitations
**Discussion**	23c	Discuss any limitations of the review processes used.	Discussion; Limitations
**Discussion**	23d	Discuss implications of the results for practice, policy, and future research.	Discussion; Conclusions
**Other information**	24a	Provide registration information for the review, including register name and registration number, or state that the review was not registered.	Materials and Methods; OSF Registries [34]
**Other information**	24b	Indicate where the review protocol can be accessed, or state that a protocol was not prepared.	Materials and Methods; OSF Registries [34]
**Other information**	24c	Describe and explain any amendments to information provided at registration or in the protocol.	Materials and Methods: targeted pre-submission update; Supplementary File S2
**Other information**	25	Describe sources of financial or non-financial support for the review and the role of the funders or sponsors.	Funding
**Other information**	26	Declare any competing interests of review authors.	Conflicts of Interest
**Other information**	27	Report which data, code, and other materials are publicly available and where they can be found.	Data Availability Statement; Supplementary File S3
